# Pernicious Anemia and Vitamin B12 Deficiency Presenting As Pseudothrombotic Microangiopathy and Developing Secondary Thrombocytopenia After Treatment: A Case Report

**DOI:** 10.7759/cureus.32095

**Published:** 2022-12-01

**Authors:** Hazem Abosheaishaa, Mahmoud Nassar, Muhammad Ghallab, Mohammed Abdelwahed, Amr Ali, Bassel Ibrahim, Eben Kimball

**Affiliations:** 1 Internal Medicine, Icahn School of Medicine at Mount Sinai, Queens Hospital Center, New York City, USA; 2 Internal Medicine/Gastroenterology, Cairo University, Cairo, EGY; 3 Internal Medicine, Icahn School of Medicine at Mount Sinai/NYC Health + Hospitals/Queens, New York City, USA; 4 Internal Medicine, Icahn School of Medicine at Mount Sinai, NYC Health and Hospitals/Queens, New York City, USA; 5 Pathology, Donald and Barbara Zucker School of Medicine at Hofstra/Northwell, Uniondale, USA; 6 Internal Medicine, University of Houston, Houston, USA; 7 Internal Medicine, Queens Hospital Center, Jamaica, USA

**Keywords:** secondary/reactive thrombocytosis, thrombotic thrombocytopenic purpura (ttp)-like syndrome, vitamin b12 deficiency anemia, pernicious anemia, pseudo ttp

## Abstract

Pernicious anemia (PA) is an autoimmune disease secondary to chronic atrophic gastritis leading to vitamin B12 deficiency. Rarely, some patients may develop advanced hematological complications that mimic those of thrombotic thrombocytopenic purpura (TTP). Differentiating these conditions is crucial because they require different management. We present a case of a 68-year-old male who presented with generalized weakness, fatigue, and shortness of breath. This patient had anemia, thrombocytopenia, and a markedly deficient serum level of vitamin B12. The symptoms initially mimicked those associated with TTP, but the activity of ADAMTS 13 was normal. A diagnosis of pseudo-TTP has been made due to vitamin B12 deficiency resulting from PA with reactive thrombocytosis. Ultimately, vitamin B12 deficiency pseudo-TTP should be considered a differential diagnosis for therapy refractory TTP because of its different management strategies ranging from parenteral Vitamin B12 in PA patients with highly favorable outcomes to more advanced treatment with less favorable outcomes in TTP patients.

## Introduction

Pernicious anemia (PA) is an autoimmune disease secondary to chronic atrophic gastritis leading to vitamin B12 deficiency [1]. The diagnosis of PA is often confirmed by the presence of anti-intrinsic factor antibodies, which, despite their low sensitivity, are highly specific [2]. During the early stages of the disease, patients are usually asymptomatic, contributing to an underestimated diagnosis rate [2]. As the disease progresses, patients develop hematological abnormalities and neuropsychiatric symptoms in more severe cases [2]. Hematological findings commonly found in peripheral blood smears include megaloblastic anemia, elevated lactate dehydrogenase (LDH), and hyper-segmented neutrophils [3]. A rare condition may result in developing more severe hematological complications, including hemolytic anemia, thrombocytopenia, and schistocytes on peripheral blood smears, which may mimic the symptoms of more severe conditions; thrombotic microangiopathies, particularly thrombotic thrombocytopenic purpura (TTP) [4,5]. Differentiating between the two conditions is critical since their management differs, ranging from parenteral vitamin B12 in patients with PA with highly favorable outcomes to more advanced treatment with less favorable outcomes in patients with TTP [3]. We present the case of a 68-year-old male who initially presented with symptoms that mimicked TTP but were ultimately diagnosed as having pseudo-TTP due to vitamin B12 deficiency in the context of PA. Following vitamin B12 treatment, he developed reactive thrombocytosis.

## Case presentation

We present a 68-year-old male with a history of alcohol use disorder who presented to the emergency department (ED) with generalized weakness, fatigue, and shortness of breath. The patient denied experiencing chest pain, palpitations, dizziness, abdominal pain, nausea, vomiting, changes in bowel habits, fever, and changes in appetite. On presentation, the patient had a blood pressure of 80/50 mmHg, a pulse rate of 95 beats per minute, a respiratory rate of 16 cycles per minute, and a body temperature of 98.6 F. The patient was alert, awake, and oriented to time, place, and person during the examination. However, He appeared jaundiced, pale, weak, and stressed. There were no apparent murmurs in S1 or S2 during the cardiac examination. During a chest examination, there are loud vesicular sounds and right lower lobe crackles. Abdominal examination revealed no hepatosplenomegaly or tenderness. A neurological examination revealed no abnormalities and no swelling in the lower extremities.

ED investigations were significant for severe anemia, thrombocytopenia, mild leukocytosis, acute kidney injury, indirect hyperbilirubinemia, transaminitis, compensated metabolic acidosis with low bicarbonate and elevated procalcitonin. Troponin, D-dimer, Lipase, and Ethanol levels were within normal range. Chest x-ray revealed right lower lobe pneumonia. (Table 1).

**Table 1:** Initial labs are done at the ED

Labs	value	Reference range
CBC		
WBCs	11.4 x10^3^/MCL	4.8-10.8 x10^3^/MCL
Hemoglobin (Hb)	4 g/dL	14-18 g/dL
Hematocrit	14.5%	42-52
MCV	120 fL	80-99
Platelets (PLT)	75x10^3^/MCL	(150-450) x10^3^/MCL
KFT’s		
BUN	30 mg/dL	6-23 mg/dL
Creatinine	1.44 mg/dL	0.5-1.2 mg/dL
LFTs		
Total Bilirubin	6.2 mg/dL	0.0-1.2 mg/dL
Direct Bilirubin	1.8 mg/dL	0.0-1.2 mg/dL
ALT	57 U/L	0-33 U/L
AST	64 U/L	5-35 U/L
Coags		
INR	1.6	
PT	18.2	1-13 seconds
ABG		
PH	7:39	7.32-7.43
PCO2	30	38-41 mmHg
HCO3	6	22-29 mEq/L
procalcitonin level	1.29	< 0.25 ng/mL

In the emergency department, the patient received two liters of IV normal saline, Piperacillin-tazobactam 3.375 g IV, Vancomycin one gram IV, Paracetamol 975 mg tablet oral, Chlordiazepoxide 50 mg IV, and Pantoprazole 40 mg IV. The patient received one packed RBC after cross-matching was confirmed. The patient was admitted to the intensive care unit (ICU) for treatment of pneumonia and severe symptomatic anemia.

After hospital admission, the patient was transfused with four packed red blood cells and continued receiving IV antibiotics and IV fluids. Further blood work for anemia revealed low serum iron, low TIBC, high Ferritin level, low serum vitamin B12 level, elevated reticulocyte percent, low reticulocyte index and normal absolute reticulocytic count (Table 2).

**Table 2:** Blood work of anemia

Lab	Value	Reference range
Serum Iron	28 mg/dL	45-165 mg/dL
TIBC	134 mg/dL	220-430 mg/dL
Ferritin	1,659	30 – 400 ng/mL
Serum Vitamin B12	150	232-1245 pg/mL
Reticulocyte %	1.74	0.5-1.5
Rticulocyte index	0.49	0.5-2.5
Absolute reticuloctic count	0.0449	0.0221-0.0963 x 10^6 ^/MCL

The abdominal US excluded biliary obstruction. Further investigations revealed LDH: 225 (n: 100-250 U/L), haptoglobin: 91 (n: 41-165) mg/dL, ADAMTS 13 activity of 63% (n: 50%-100%) and schistocytes in the peripheral blood smear. Anti-intrinsic factor antibody was significantly high at 218.6 AU/ML (n: 0.0-1.1), while MMA and homocysteine were not collected. This patient was diagnosed with PA based on the combination of low serum vitamin B12 and high anti-intrinsic factor antibody levels. Vitamin B12 was prescribed at a high dose of 1,000 ug iv daily in the fifth day of admission. After stabilizing his condition, the patient was transferred to the floor for further management. The patient was transferred from the ICU to a ward. Follow-up labs were explained in Table 3.

**Table 3:** Follow-up CBC results after vitamin B12 correction

CBC	ED	1^st^ day	4^th^ day	9^th^ day	10^th^ day	11^th^ day	12^th^ day	13^th^ day	18^th^ day	Reference
WBCs	11.4	10.87	12.05	14.26	12.87	10.22	10.22	11.69	9.29	4.8-10.8
Hb	4	10.3	11.7	11.8	11.71	10.5	11.3	10.7	11.2	14-18
PLT	75	329	831	12	1137	1075	936	911	509	150-450

In the context of PA with reactive thrombocytosis after vitamin B12 correction, the patient was diagnosed with pseudo-thrombotic microangiopathy due to vitamin B12 deficiency Following the stabilization of the patient's condition, the patient was discharged from the hospital and instructed to follow up with the outpatient hematology clinic.

## Discussion

TTP is characterized by acute hemolytic anemia, thrombocytopenia, bleeding factors, depletion, and organ damage. Vitamin B12 deficiency is associated with similar criteria [6]. Vitamin B12 plays a significant role in the bone marrow's maturation of red blood cells. Decreased levels of B12 can result in macrocytic hypochromic anemia, which leads to the destruction of peripheral blood RBCs. Vitamin B12 deficiency is associated with a wide range of hematologic manifestations. Symptoms of vitamin B12 deficiency include anemia, leukopenia, thrombocytopenia, hyper-segmented neutrophils, and pancytopenia. The incidence of pseudo-TTP is estimated to be 2.5% [7].

Andres et al. [8] studied the possible hematological complications of vitamin B12 deficiency in 201 patients and described “pseudo” thrombotic microangiopathy in 2.5 % of the studied populations. Tadakamalla et al. reported a case of a 31-year-old female with a history of fatigue and paresthesia in both legs for one week. Indirect hyperbilirubinemia, thrombocytopenia, and schistocytes were found with subacute onset of fatigue and paresthesia, and TTP was suspected. However, platelet count did not improve with four days of plasmapheresis. Instead, after the correction of low vitamin B12, hematological complications improved, and more investigations revealed positive anti-intrinsic factor antibodies, which confirmed the diagnosis of PA associated pseudo-TTP [6].

A case series have noted that pseudo-thrombotic microangiopathy is associated with prolonged PT, low fibrinogen and platelets, schistocytes, and high LDH and D-dimer levels, as well as multi-organ dysfunction [9]. In 2017, Kandel et al. reported a case of PA presented with multi-organ dysfunction syndrome, diagnosed later as pseudo-TTP [10].

The distinction between pseudo-TTP and TTP can be challenging. Both conditions may present with hemolytic anemia, thrombocytopenia, and schistocytosis. By distinguishing between the two conditions, patients with pseudo-TTP will avoid unnecessary interventions, and plasma transfusion complications can be reduced, as pseudo-TTP does not respond to plasma exchange. [11].

Due to the therapeutic dilemma in cases of TTP, which is a serious condition that needs rapid intervention with plasma exchange therapy [12], suggesting a set of routine labs to be done, including B12, methylmalonic acid level, and reticulocyte count, to help distinguish TTP from Vitamin B12 associated pseudo-TTP.

It is essential to realize that while plasma exchange can temporarily relieve symptoms, vitamin B12 is the main line of treatment. In response to vitamin B12 replacement, hemolysis markers typically decrease after one to two days, and reticulocytes increase after three to four days. In 85% of the cases, there was a complete response to parenteral B12 replacement within 14 days, while in 15% of cases, it took up to six months for the patient to achieve a complete response [13].

Reactive thrombocytosis for vitamin B12 can happen. Ogston et al. described reactive thrombocytosis in response to treating thrombocytopenia and anemia. It should get back to normal within a reasonable timeline [13]. Blood tests such as fibrinogen, hyperlipidemia, and D-dimer should also be evaluated to fully assess the patient's viscosity situation. The plasma viscosity was not significantly different between groups despite significant differences in platelet counts, according to Toprak et al. [14]. Due to the lack of a treatment requirement, awareness of the possibility of thrombocytosis following vitamin B12 replenishment should be raised. Close monitoring and observation are the only requirements. 

## Conclusions

Vitamin B12 deficiency pseudo-TTP is a phenomenon that should be kept as a differential diagnosis of therapy refractory TTP. Reactive thrombocytosis as a response to cobalamin therapy can be markedly high and needs further evaluation. Still, other inflammatory and bleeding markers are required for a complete picture of the patient's homeostasis.
